# Transfusion ratios and survival in severe blunt trauma patients receiving massive transfusion

**DOI:** 10.1038/s41598-025-11338-7

**Published:** 2025-07-15

**Authors:** Toru Takiguchi, Tomohisa Seki, Takashi Tagami, Yu Akagi, Ryuta Nakae, Hiromasa Ito, Yoshimasa Kawazoe, Ichiro Okada, Shiei Kim, Masaaki Inoue, Kazuhiko Ohe, Shoji Yokobori

**Affiliations:** 1 Department of Emergency and Critical Care Medicine, Nippon Medical School, 1-1-5 Sendagi, Bunkyo-ku, Tokyo, Japan; 2 Department of Healthcare Information Management, The University of Tokyo Hospital, Tokyo, Japan; 3 Department of Emergency and Disaster Medicine, The Jikei University School of Medicine, Tokyo, Japan; 4 Department of Biomedical Informatics, Graduate School of Medicine, The University of Tokyo, Tokyo, Japan; 5 Artificial Intelligence and Digital Twin in Healthcare, Graduate School of Medicine, The University of Tokyo, Tokyo, Japan

**Keywords:** Transfusion ratio, Blunt trauma, Massive transfusion, Clustering, Truncal trauma, Shock, Outcomes research, Trauma, Surgery

## Abstract

The optimal transfusion ratios for severe blunt trauma requiring massive transfusion remain unclear. This nationwide retrospective cohort study used data from the Japan Trauma Data Bank (2019–2022) and included patients receiving ≥ 10 units of packed red blood cells (pRBC) within 24 h. The fresh frozen plasma (FFP)-to-pRBC and platelet concentrate (PC)-to-pRBC ratios were categorized as 0–0.5, 0.5–1, 1–1.5, 1.5–2, and > 2. Among 2,849 eligible patients, an FFP-to-pRBC ratio of 1–1.5 was associated with significantly higher in-hospital survival than 0.5–1 (adjusted odds ratio [OR], 1.46; 95% confidence interval [CI], 1.12–1.92; *P* = 0.006). A PC-to-pRBC ratio of 1.5–2 also showed a trend toward improved survival (1.62; 1.00–2.69; *P* = 0.053). Patients were categorized into three phenotypes: truncal trauma with shock (70.3%), moderate head and extremity trauma (11.8%), and severe head trauma with consciousness disturbances (17.9%). In the truncal trauma with shock phenotype, FFP-to-pRBC ratios of 1–1.5 (1.56; 1.12–2.20; *P* = 0.010) and > 2 (2.32; 1.14–5.10; *P* = 0.027) were associated with improved survival. Higher FFP-to-pRBC and PC-to-pRBC ratios may be associated with improved survival, especially in truncal trauma with shock.

**Supplementary Information:**

The online version contains supplementary material available at 10.1038/s41598-025-11338-7.

## Introduction

Severe trauma poses a significant challenge to global public health. According to the Global Burden of Diseases, Injuries, and Risk Factors Study, trauma is responsible for approximately 8% of all deaths each year^1^. Post-traumatic bleeding and the resulting traumatic coagulopathy continue to be major contributors to potentially preventable multiorgan failure and mortality^2^. Recent clinical guidelines recommend that massive transfusion protocols for the initial management of major haemorrhage emphasise the maintenance of a high fresh frozen plasma (FFP)-to-packed red blood cells (pRBC) ratio and high platelet concentrate (PC)-to-pRBC ratio to achieve haemostasis and effectively manage trauma-induced coagulopathy^3–5^.

The optimal transfusion ratio for massive transfusion protocols in patients with trauma remains unknown. The PROPPR trial, a landmark randomised controlled study on massive transfusion ratios, demonstrated that using a 1:1:1 ratio of FFP, PC, and pRBC resulted in quicker haemostasis and reduced 24-hour mortality due to exsanguination compared with using a 1:1:2 ratio, although it did not decrease 30-day mortality^6^. Literature reviews on massive transfusion suggest that protocols should use ratios of 1:1:1 or 1:1:2 for FFP, PC, and pRBC^7–25^. Current guidelines in Europe, the United States, and Japan recommend a massive transfusion protocol with high FFP-to-pRBC and PC-to-pRBC ratios ranging from 1:2 to 1:1^3–5^. However, clinical studies forming the basis of current guidelines have not examined transfusion ratios > 1 for both FFP-to-pRBC and PC-to-pRBC. Furthermore, the optimal range for high transfusion ratios (> 1) has not been determined.

Although severe blunt trauma encompasses a spectrum of coagulopathic phenotypes^26–30^, the optimal transfusion ratio for different trauma phenotypes remains unclear. Coagulopathy associated with traumatic injury is caused by multiple factors and complex interactions. Trauma-induced coagulopathy is associated with the severity of injury, shock, hypothermia, and haemodilution^26–30^. Furthermore, a retrospective observational study using large-scale registry data demonstrated that specific combinations of multiple injuries significantly affected patient outcomes^31^. In addition, blunt trauma typically results in more extensive tissue injury and hypoperfusion compared to penetrating trauma, leading to more pronounced coagulopathy^26,30,32^. Consequently, the optimal transfusion ratio for managing specific phenotypes of blunt trauma may vary. Unsupervised agglomerative clustering, a technique that identifies patient groups based on multiple variables without prior assumptions, shows promise for refining phenotype classification in blunt trauma^33–38^. Applying machine learning to sub-phenotypic blunt trauma may ultimately enable the development of more targeted transfusion strategies, potentially improving the outcomes for specific trauma phenotypes.

The aim of the present study was to evaluate the optimal transfusion ratio > 1 for massive transfusion in severe blunt trauma in both an entire cohort and specific phenotypes, using a nationwide trauma registry in Japan.

## Methods

### Ethical approval

This study was approved by the Institutional Review Board of Nippon Medical School Hospital (B-2024-896). Due to the retrospective nature of the study, informed consent was waived by the Institutional Review Board of Nippon Medical School Hospital. All methods were performed in accordance with the relevant guidelines and regulations.

### Data source

This retrospective observational study used data from the Japan Trauma Data Bank (JTDB), a prospective multicentre nationwide trauma registry^39^. Established in 2003 by the Trauma Registry Committee of the Japanese Association for the Surgery of Trauma and the Committee for Clinical Care Evaluation of the Japanese Association for Acute Care Medicine, the JTDB is managed by Japan Trauma Care and Research to improve and ensure the quality of trauma care in Japan. The JTDB requires the registration of all severe trauma cases with an Abbreviated Injury Scale (AIS) score ≥ 3^40^; however, registration of all patients is also permitted. This database contains information on 303 facilities across Japan, compiled annually^39^. The JTDB includes patient characteristics, injury type, mechanism, vital signs, AIS score, injury severity score (ISS)^41^, revised trauma score (RTS)^42^, trauma and injury severity score and probability of survival (TRISS-PS)^43^, in-hospital treatment and procedures, and outcomes. The data collection items in this registry were revised in 2019, with the amount of blood transfused within 24 h added as a new entry. In this study, we used JTDB 2019 registry cases following the implementation of new data collection items.

### Study population

This study included trauma patients registered in the JTDB dataset between January 2019 and December 2022. The exclusion criteria were as follows: (1) penetrating injuries; (2) burns; (3) mixed injuries; (4) other non-blunt trauma injuries; (5) Unknown; (6) non-direct transportation; (7) cardiac arrest on hospital arrival; (8) patients who did not receive a transfusion within 24 h; and (9) patients who received < 10 units of pRBCs within 24 h. The final study population consisted of patients who received massive transfusions following blunt trauma, defined as the administration of ≥ 10 units of pRBCs within 24 h.

### Data collection and outcome

The following patient data were collected from the JTDB database: age, sex, Charlson Comorbidity Index (CCI)^44^, vital signs on hospital arrival (systolic blood pressure, heart rate, respiratory rate, body temperature, and Glasgow Coma Scale), maximum AIS region score (head and neck, face, chest, abdomen, extremities, and external), ISS, RTS, TRISS-PS, amount of blood products within 24 h (pRBC, FFP, and PC), treatments (the use of tranexamic acid and vasopressors) and procedures (resuscitative endovascular balloon occlusion of the aorta and transcatheter arterial embolization) in the emergency department, in-hospital surgery, and outcomes (in-hospital survival). Massive transfusion was defined as the administration of ≥ 10 units of pRBC within 24 h. (In Japan, 1 unit of pRBC is approximately 120 m.) The primary outcome was in-hospital survival owing to any cause.

### Statistical analyses

#### Clustering method

Clustering variables were selected based on a previous study using the JTDB which analysed trauma clinical phenotypes and biological profiles related to inflammation and coagulation disorders as follows: age, sex, CCI, vital signs on hospital arrival (systolic blood pressure, heart rate, respiratory rate, body temperature, and Glasgow Coma Scale score), and maximum AIS region scores (head and neck, face, chest, abdomen, extremities, and external)^45^. To handle the missing data for clustering, the specific imputation methods were chosen based on the type of variable. Predictive mean matching was used for continuous variables such as age, CCI, systolic blood pressure, heart rate, respiratory rate, body temperature, Glasgow Coma Scale score, and maximum AIS region scores. A logistic regression model was used for binary variables such as sex. A distance matrix was calculated using Euclidean distance, followed by an elbow plot to determine the optimal number of clusters based on the within-cluster sum of squares. Finally, agglomerative hierarchical clustering was performed using Ward’s minimum variance method. A heatmap of the accompanying data was generated to visualise the clustering results. Missing data were imputed prior to calculating the distance matrix and performing hierarchical clustering; however, the imputed data were not used for cluster comparisons or subsequent statistical analyses. Although a previous study identified 11 clinical phenotypes using similar variables^45^, we independently performed clustering for our study population using the elbow method to determine the number of clusters. Thus, the phenotypes in this study were newly derived and not selected from prior classifications.

#### Statistical testing

Continuous variables were presented as mean values with standard deviations. Categorical variables were presented as frequencies and percentages. We compared baseline characteristics across phenotypes using analysis of variance or Kruskal-Wallis tests, as appropriate, for continuous variables and the chi-square tests for categorical variables. The survival rates for each FFP-to-pRBC and PC-to-pRBC ratios were presented for the total cohort and by phenotype, along with the number of survivors out of the total number of patients in each FFP-to-pRBC ratio category. Based on literature review and clinical guidelines of massive transfusion, which suggest that massive transfusion should employ FFP, PC, and pRBC ratios between 1:1:1 and 1:1:2, we classified the FFP-to-pRBC and PC-to-pRBC ratios into the following categories: 0–0.5 (including 0.5), 0.5–1 (including 1), 1–1.5 (including 1.5), 1.5–2 (including 2), and > 2^3–5,7–10^. Multivariable logistic regression analyses were conducted to assess the association between FFP-to-pRBC and PC-to-pRBC ratios and in-hospital survival. The covariates used in clustering were adjusted for age, sex, CCI, vital signs on hospital arrival (systolic blood pressure, heart rate, respiratory rate, body temperature, and Glasgow Coma Scale), and maximum AIS region score (head and neck, face, chest, abdomen, extremities, and external). The reference category for the multivariable logistic regression analyses of FFP-to-pRBC and PC-to-pRBC ratios was greater than 0.5–1 (including 1), based on the recommended transfusion ratios from literature reviews and clinical guidelines on massive transfusion^3–5,7–10^. Data were reported as odds ratios (ORs) with 95% confidence intervals (CIs). All statistical analyses were performed using R software package version 4.3.1 (R Foundation for Statistical Computing, Vienna, Austria). Two-sided values of *p* < 0.05 were considered statistically significant.

## Results

**Fig. 1:**
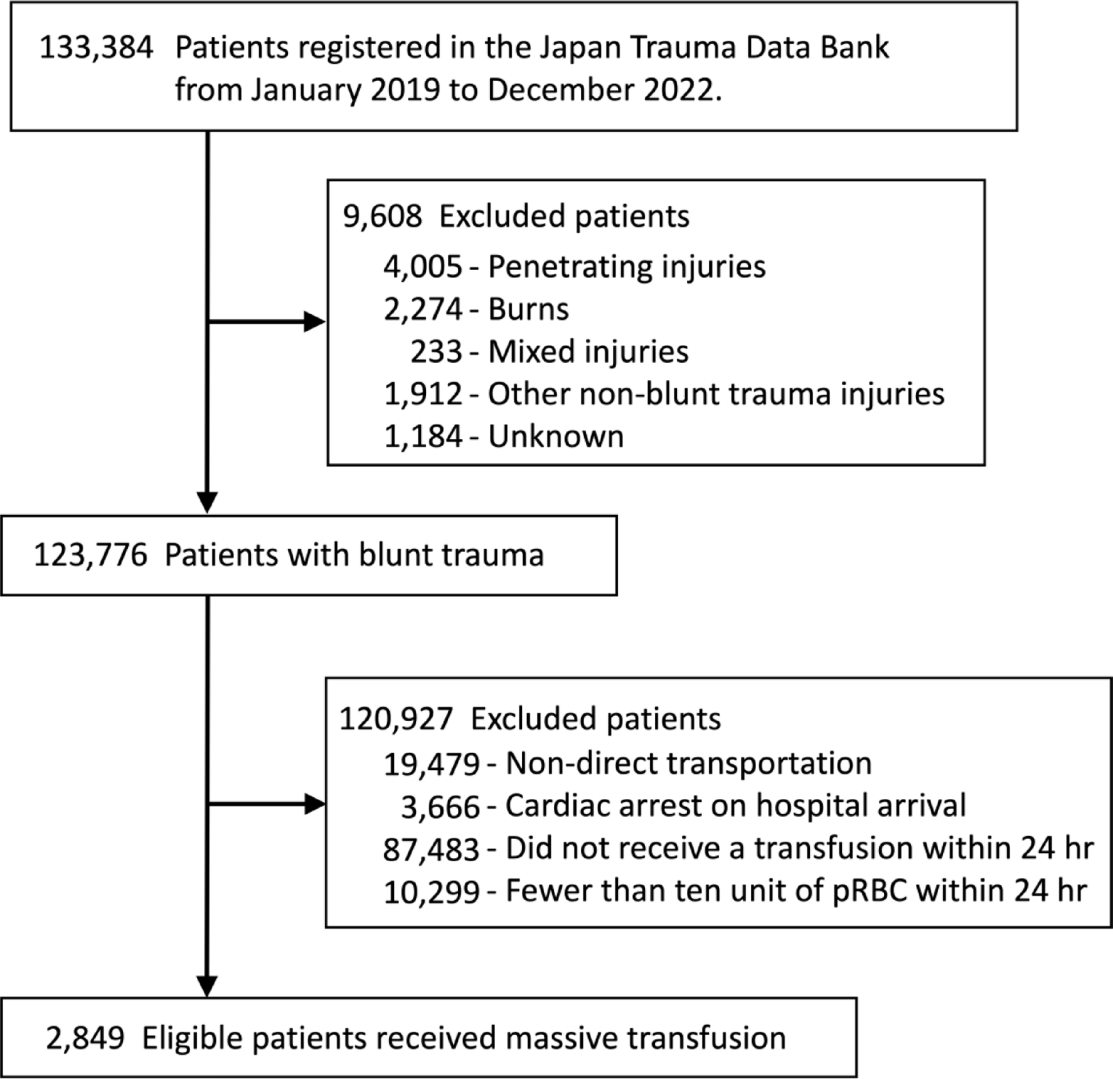
Patient selection.

Overall, 133,384 patients with trauma were registered with the JTDB during the study period. We identified 2,849 patients with blunt trauma who underwent massive transfusions (Fig. 1).

**Fig. 2:**
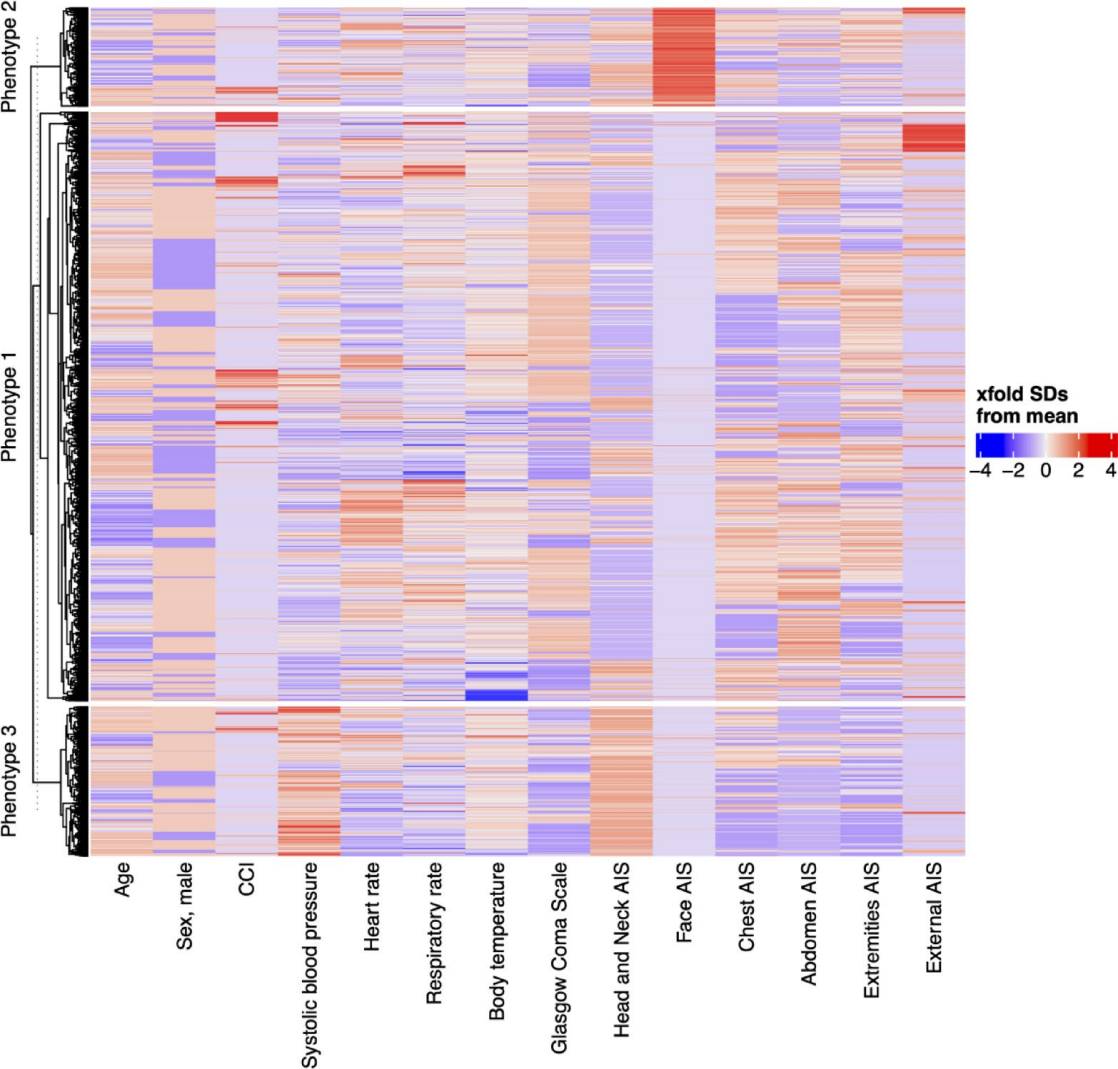
Heatmap and dendrogram for visualization of clustering results. SDs, standard deviations; CCI, Charlson Comorbidity Index; AIS, Abbreviated Injury Scale.

Subsequently, patients were categorised into three phenotypes using unsupervised agglomerative clustering (Fig. 2 and Supplementary Fig. S1 online).

Table 1 compares the baseline characteristics of the entire cohort with those of the three phenotypes. Phenotype 1 (*n* = 2,004 [70.3%]), which comprised the majority of patients, included those with truncal trauma involving the chest, abdomen, and extremities, along with shock characterised by low systolic blood pressure and high heart rate (in-hospital survival: 72.2%). Phenotype 2 (*n* = 336 [11.8%]) included patients with moderate head and extremity trauma, moderate consciousness disturbance characterised by a low Glasgow Coma Scale score, and shock with low systolic blood pressure and high heart rate (in-hospital survival: 72.6%). Phenotype 3 (*n* = 509 [17.9%]) included patients with severe head trauma and severe consciousness disturbances, as indicated by a low Glasgow Coma Scale score (in-hospital survival: 48.5%). The mean (SD) Glasgow Coma Scale score was 9 (4.4) for phenotype 2 and 7 (4.1) for phenotype 3, with a significant difference between the two phenotypes (*P* < 0.001) (see Supplementary Fig. S2 online).

**Fig. 3:**
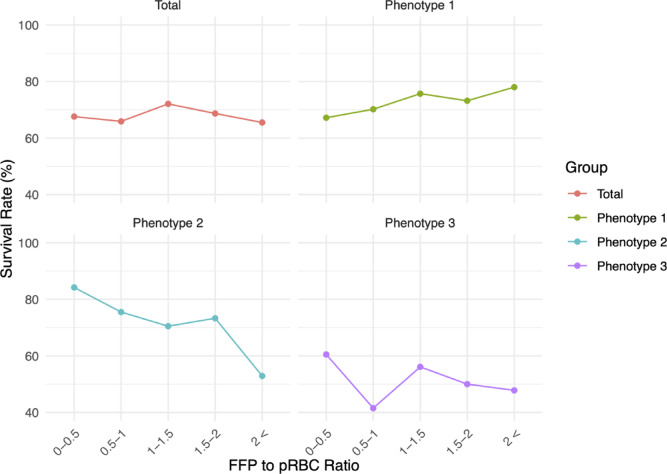
Survival rate by FFP-to-pRBC ratio for each group. FFP, fresh frozen plasma; pRBC, packed red blood cells.

Table 2 shows the survival rate and number of survivors out of the total number of patients for each FFP-to-pRBC ratio. The highest survival rate in each FFP-to-pRBC ratio category was observed at a ratio of 1–1.5 in the total cohort, > 2 in phenotype 1, and 0–0.5 in phenotypes 2 and 3 (Fig. 3). The largest number of patients in each FFP-to-pRBC ratio category was in the 0.5–1 ratio for the total cohort and across all three phenotypes. The total number for each FFP-to-pRBC ratio category can be found Supplementary Table S1 online.

**Fig. 4:**
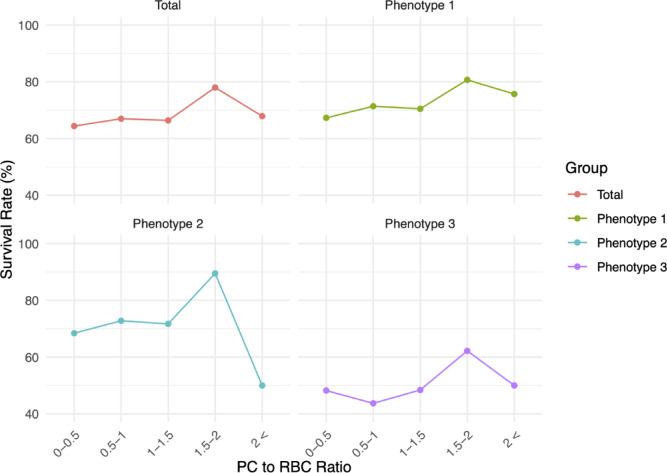
Survival rate by PC-to-pRBC ratio for each group. PC, platelet concentrate; pRBC, packed red blood cells.

Table 3 shows the survival rate and number of survivors out of the total number of patients for each PC-to-pRBC ratio. The highest survival rate in each PC-to-pRBC ratio category was observed at a ratio of 1.5–2 in the total cohort and across all three phenotypes (Fig. 4). The largest number of patients was in the 0.5–1 ratio for the total cohort and across all three phenotypes.

Table 4 shows the multivariate logistic regression analyses of in-hospital survival based on the FFP-to-pRBC ratio for the entire cohort and across the three phenotypes. In the total cohort, a ratio of 1–1.5 was associated with a significantly higher in-hospital survival rate compared to a ratio of 0.5–1 (adjusted OR = 1.46; 95% CI, 1.12–1.92; *P* = 0.006). For phenotype 1, ratios of 1–1.5 (adjusted OR = 1.56; 95% CI, 1.12–2.20; *P* = 0.010) and > 2 (adjusted OR = 2.32; 95% CI, 1.14–5.10; *P* = 0.027) were associated with a significantly higher in-hospital survival rate compared to a ratio of 0.5–1.

Table 5 shows the multivariate logistic regression analyses of in-hospital survival based on the PC-to-pRBC ratio for the entire cohort and across the three phenotypes. In the total cohort, although not statistically significant, a ratio of 1.5–2 was associated with a higher in-hospital survival rate compared to a ratio of 0.5–1 (adjusted OR = 1.62; 95% CI, 1.00–2.69; *P* = 0.053). Across the three phenotypes, multivariate logistic regression analyses revealed no significant differences for in-hospital survival.

**Table 1:** Comparison of the baseline characteristics for the total cohort and across the three phenotypes.^a^.

Variables	Total *n* = 2,849		missing data		Phenotype 1 *n* = 2,004		missing data		Phenotype 2 *n* = 336		missing data		Phenotype 3 *n* = 509		missing data		*P* value
Age, years	58	(22.4)	1	(0.0)	58	(22.3)	1	(0.0)	52	(23.0)	0	(0)	63	(21.2)	0	(0)	< 0.001
Sex, male	1,866	(65.8)	14	(0.0)	1,229	(61.7)	11	(0.5)	245	(72.9)	0	(0)	392	(77.5)	3	(0.6)	< 0.001
Charlson comorbidity index	0.42	(0.99)	0	(0)	0.46	(1.08)	0	(0)	0.33	(0.76)	0	(0)	0.33	(0.68)	0	(0)	0.007
Vital signs																	
Systolic blood pressure	105	(41.0)	82	(2.9)	95	(35.0)	62	(3.1)	105	(39.5)	10	(3.0)	145	(39.2)	10	(2.0)	< 0.001
Heart rate	103	(28.9)	60	(2.1)	104	(28.7)	48	(2.4)	110	(28.0)	4	(1.2)	96	(29.1)	8	(1.6)	< 0.001
Respiratory rate	24	(9.2)	269	(9.4)	25	(9.6)	187	(9.3)	24	(7.8)	33	(9.8)	21	(7.6)	49	(9.6)	< 0.001
Body temperature (°C)	36.0	(1.20)	499	(17.5)	35.9	(1.28)	344	(17.2)	36.0	(1.01)	62	(18.5)	36.2	(0.80)	93	(18.3)	< 0.001
Glasgow Coma Scale	10	(4.6)	70	(2.5)	11	(4.4)	50	(2.5)	9	(4.4)	3	(0.9)	7	(4.1)	17	(3.3)	< 0.001
Abbreviated injury scale																	
Head and Neck	2	(2.1)	62	(2.2)	1	(1.8)	41	(2.0)	3	(1.8)	7	(2.1)	4	(1.0)	14	(2.8)	< 0.001
Face	0	(0.7)	62	(2.2)	0	(0.2)	41	(2.0)	2	(0.6)	7	(2.1)	0	(0.3)	14	(2.8)	< 0.001
Chest	2	(1.7)	62	(2.2)	2	(1.7)	41	(2.0)	2	(1.6)	7	(2.1)	2	(1.7)	14	(2.8)	< 0.001
Abdomen	2	(1.6)	62	(2.2)	2	(1.6)	41	(2.0)	1	(1.5)	7	(2.1)	1	(1.2)	14	(2.8)	< 0.001
Extremities	2	(1.7)	62	(2.2)	3	(1.7)	41	(2.0)	3	(1.6)	7	(2.1)	1	(1.4)	14	(2.8)	< 0.001
External	0	(0.7)	62	(2.2)	0	(0.7)	41	(2.0)	0	(0.6)	7	(2.1)	0	(0.4)	14	(2.8)	< 0.001
Injury severity scale	29	(13.3)	90	(3.2)	29	(13.7)	62	(3.1)	35	(13.0)	10	(3.0)	30	(11.3)	18	(3.5)	< 0.001
Revised trauma score	6.0	(1.8)	341	(12.0)	6.2	(1.8)	235	(11.7)	5.7	(1.7)	41	(11.9)	5.6	(1.5)	65	(12.8)	< 0.001
TRISS PS, %	71.6	(29.7)	415	(14.6)	74.2	(29.9)	285	(14.2)	67.0	(30.6)	50	(14.9)	64.0	(26.9)	80	(15.7)	< 0.001
Blood transfusion within 24 h																	
pRBC, units	20	(14.2)	0	(0)	20	(14.2)	0	(0)	20	(13.8)	0	(0)	18	(14.3)	0	(0)	0.007
FFP, units	23	(41.5)	86	(3.0)	23	(36.1)	50	(2.5)	23	(16.7)	11	(3.3)	23	(66.1)	25	(4.9)	0.94
PC, units	16	(14.7)	815	(28.6)	16	(14.9)	545	(27.2)	18	(14.7)	110	(32.7)	15	(13.5)	160	(31.4)	0.20
Treatments in emergency department																	
Tranexamic acid	1,284	(45.1)	0	(0)	894	(44.6)	0	(0)	180	(53.6)	0	(0)	210	(41.3)	0	(0)	0.002
Vasopressors	879	(30.9)	0	(0)	659	(32.9)	0	(0)	100	(29.8)	0	(0)	120	(23.6)	0	(0)	< 0.001
Procedures in emergency department																	
REBOA	413	(14.5)	0	(0)	350	(17.5)	0	(0)	36	(10.7)	0	(0)	27	(5.3)	0	(0)	< 0.001
TAE	1,565	(54.9)	0	(0)	1110	(55.4)	0	(0)	156	(46.4)	0	(0)	299	(58.7)	0	(0)	< 0.002
In-hospital surgery																	
Head surgery	533	(18.7)	0	(0)	156	(7.8)	0	(0)	62	(18.5)	0	(0)	315	(61.9)	0	(0)	< 0.001
Face surgery	70	(2.5)	0	(0)	14	(0.7)	0	(0)	51	(15.2)	0	(0)	5	(1.0)	0	(0)	< 0.001
Cervical surgery	95	(3.3)	0	(0)	50	(2.5)	0	(0)	18	(5.4)	0	(0)	27	(5.3)	0	(0)	< 0.001
Chest surgery	381	(13.4)	0	(0)	299	(14.9)	0	(0)	38	(11.3)	0	(0)	44	(8.6)	0	(0)	< 0.001
Abdominal surgery	838	(29.4)	0	(0)	707	(35.3)	0	(0)	78	(23.2)	0	(0)	53	(10.4)	0	(0)	< 0.001
Orthopedic surgery	1,326	(46.5)	0	(0)	1053	(52.5)	0	(0)	178	(53.0)	0	(0)	95	(18.7)	0	(0)	< 0.001
In-hospital survival	1,937	(68.0)	0	(0)	1446	(72.2)	0	(0)	244	(72.6)	0	(0)	247	(48.5)	0	(0)	< 0.001

^a^Data are presented as mean (standard deviation) for continuous variables and as N (percentage) for categorical variables.

TRISS PS, trauma and injury severity score probability of survival; pRBC, packed red blood cells; FFP, fresh frozen plasma; PC, platelet concentrate; REBOA, resuscitative endovascular balloon occlusion of the aorta; TAE, transcatheter arterial embolizatio.

**Table 2:** The survival rate and the number of survivors for each FFP-to-pRBC ratio category.

FFP-to-pRBC ratio	Survival rate, %	(*n* = survivor/total)
Total		
0–0.5	67.6	(150/222)
0.5–1	65.9	(795/1,206)
1–1.5	72.1	(588/816)
1.5–2	68.7	(257/374)
2 <	65.5	(95/145)
Phenotype 1		
0–0.5	67.2	(111/165)
0.5–1	70.2	(605/862)
1–1.5	75.7	(445/588)
1.5–2	73.2	(188/257)
2 <	78.0	(64/82)
Phenotype 2		
0–0.5	84.2	(16/19)
0.5–1	75.5	(105/139)
1–1.5	70.5	(74/105)
1.5–2	73.3	(33/45)
2 <	52.9	(9/17)
Phenotype 3		
0–0.5	60.5	(23/38)
0.5–1	41.5	(85/205)
1–1.5	56.1	(69/123)
1.5–2	50.0	(36/72)
2 <	47.8	(22/46)

FFP, fresh frozen plasma; pRBC, packed red blood cells.

FFP-to-pRBC ratios were categorized as follows: 0–0.5 (including 0.5), 0.5–1 (including 1), 1–1.5 (including 1.5), 1.5–2 (including 2), and > 2.

**Table 3:** The survival rate and the number of survivors for each PC-to-pRBC ratio category.

PC-to-RBC ratio	Survival rate, %	(*n* = survivor/total)
Total		
0–0.5	64.4	(446/693)
0.5–1	67.0	(516/770)
1–1.5	66.4	(217/327)
1.5–2	78.0	(149/191)
2 <	67.9	(36/53)
Phenotype 1		
0–0.5	67.3	(341/507)
0.5–1	71.4	(402/563)
1–1.5	70.5	(153/217)
1.5–2	80.7	(109/135)
2 <	75.7	(28/37)
Phenotype 2		
0–0.5	68.4	(52/76)
0.5–1	72.8	(59/81)
1–1.5	71.7	(33/46)
1.5–2	89.5	(17/19)
2 <	50.0	(2/4)
Phenotype 3		
0–0.5	48.2	(53/110)
0.5–1	43.7	(55/126)
1–1.5	48.4	(31/64)
1.5–2	62.2	(23/37)
2 <	50.0	(6/12)

PC, platelet concentrate; pRBC, packed red blood cells.

PC-to-pRBC ratios were categorized as follows: 0–0.5 (including 0.5), 0.5–1 (including 1), 1–1.5 (including 1.5), 1.5–2 (including 2), and > 2.

**Table 4:** Multivariable logistic regression analyses for in-hospital survival based on the FFP-to-pRBC ratio.

FFP-to-pRBC ratio	Adjusted OR	(95%CI)	*P* value
Total			
0–0.5	1.17	(0.74 to 1.86)	0.51
0.5–1	Reference		
1–1.5	1.46	(1.12 to 1.92)	0.006
1.5–2	1.35	(0.96 to 1.90)	0.088
2 <	1.50	(0.93 to 2.46)	0.099
Phenotype 1			
0–0.5	0.85	(0.50 to 1.48)	0.56
0.5–1	Reference		
1–1.5	1.56	(1.12 to 2.20)	0.010
1.5–2	1.46	(0.96 to 2.24)	0.081
2 <	2.32	(1.14 to 5.10)	0.027
Phenotype 2			
0–0.5	3.06	(0.40 to 66.27)	0.35
0.5–1	Reference		
1–1.5	1.16	(0.51 to 2.67)	0.72
1.5–2	2.73	(0.75 to 11.92)	0.15
2 <	0.54	(0.12 to 2.57)	0.43
Phenotype 3			
0–0.5	2.26	(0.92 to 5.74)	0.079
0.5–1	Reference		
1–1.5	1.60	(0.88 to 2.95)	0.13
1.5–2	1.07	(0.51 to 2.24)	0.86
2 <	1.37	(0.59 to 3.18)	0.46

FFP, fresh frozen plasma; pRBC, packed red blood cells; OR, odds ratio; CI, confidence interval.

FFP-to-pRBC ratios were categorized as follows: 0–0.5 (including 0.5), 0.5–1 (including 1), 1–1.5 (including 1.5), 1.5–2 (including 2), and > 2.

**Table 5:** Multivariable logistic regression analyses for in-hospital survival based on the PC-to-pRBC ratio.

PC to pRBC ratio	Adjusted OR	(95%CI)	*P* value
Total			
0–0.5	0.83	(0.62 to 1.13)	0.24
0.5–1	Reference		
1–1.5	0.97	(0.67 to 1.40)	0.86
1.5–2	1.62	(1.00 to 2.69)	0.053
2 <	1.07	(0.49 to 2.46)	0.87
Phenotype 1			
0–0.5	0.69	(0.47 to 0.99)	0.045
0.5–1	Reference		
1–1.5	1.00	(0.63 to 1.61)	0.99
1.5–2	1.29	(0.71 to 2.40)	0.42
2 <	1.22	(0.43 to 4.06)	0.73
Phenotype 2			
0–0.5	1.25	(0.42 to 3.75)	0.69
0.5–1	Reference		
1–1.5	2.74	(0.75 to 11.15)	0.14
1.5–2	5.51	(0.73 to 120.02)	0.15
2 <	0.15	(0.0053 to 2.80)	0.20
Phenotype 3			
0–0.5	1.25	(0.62 to 2.50)	0.53
0.5–1	Reference		
1–1.5	0.61	(0.27 to 1.38)	0.24
1.5–2	1.88	(0.71 to 5.19)	0.21
2 <	1.33	(0.28 to 6.67)	0.72

PC, platelet concentrate; pRBC, packed red blood cells; OR, odds ratio; CI, confidence interval.

PC to pRBC ratios were categorized as follows: 0–0.5 (including 0.5), 0.5–1 (including 1), 1–1.5 (including 1.5), 1.5–2 (including 2), and > 2.

## Discussion

 This nationwide cohort study demonstrated that FFP-to-pRBC and PC-to-pRBC transfusion ratios > 1 were associated with improved survival rates in patients with severe blunt trauma undergoing massive transfusion. Ratios of 1 to 1.5 for FFP-to-pRBC and 1.5 to 2 for PC-to-pRBC were particularly effective for enhancing in-hospital survival. Notably, in patients classified as having phenotype 1 (truncal trauma with shock), the association with improved survival was even more pronounced with higher FFP-to-pRBC transfusion ratios, suggesting that specific trauma phenotypes may benefit from tailored transfusion strategies.

While prior research, including the PROPPR trial, primarily focused on transfusion ratios up to 1:1, our study examined transfusion ratios > 1 for both FFP-to-pRBC and PC-to-pRBC^6^. Several studies comparing high ratios (≥ 1) with low ratios (< 1) for FFP-to-pRBC and PC-to-pRBC in massive transfusion have shown that high ratios improved survival^46–48^. However, in these studies, the 1:1 ratio, already recommended by the PROPPR trial and clinical guidelines, was included in the high-ratio group^6–10^. Although a recent retrospective study showed that an FFP-to-RBC ratio > 1 was associated with favourable survival^49^, the optimal transfusion ratio remains unclear. Our study addressed this knowledge gap, suggesting that FFP-to-pRBC ratios > 1 and ≤ 1.5, and PC-to-pRBC ratios > 1.5 and ≤ 2, were particularly effective in enhancing in-hospital survival.

A high FFP-to-pRBC ratio was significantly associated with improved in-hospital survival rates, especially in patients with truncal trauma with shock (phenotype 1). Truncal trauma leads to extensive tissue damage, hypoperfusion, and shock, quickly depleting coagulation factors and causing systemic coagulopathy, which a high FFP ratio can effectively counter^28–30^. In contrast, in head trauma, where trauma-induced coagulopathy is often more severe, a high FFP-to-pRBC ratio alone may not be sufficient to fully restore coagulation factors^27,50–52^. Additionally, the increased volume due to a high FFP ratio can increase intracranial pressure, potentially worsening intracranial haemorrhage. The observed survival benefit associated with higher FFP-to-pRBC ratios may be explained by the pathophysiological mechanisms discussed above, and we believe this supports their potential clinical utility, particularly for patients showing truncal trauma with shock.

Our study had several limitations. First, this was a retrospective observational study, which may have introduced bias due to unmeasured confounding factors. Second, there are concerns regarding external validity as the data were derived exclusively from Japanese patients. Consequently, the findings may not be generalisable to other populations because of variations in trauma care practices, patient demographics, and healthcare systems across different countries and regions. Third, the database did not specify the cause of death, preventing analyses of cause-specific mortality. Fourth, although definition of massive transfusion as ≥ 3 units of pRBC per hour is considered a more contemporary and accurate approach to minimize survival bias, the JTDB does not record timestamps for individual transfusion events; this precludes calculation of hourly transfusion rates. Fifth, the JTDB does not include data on the administered volumes of crystalloids and artificial colloids. These variables may influence patient outcomes. Sixth, transfusion ratios were retrospectively calculated based on 24-h cumulative volumes; therefore, they do not reflect real-time changes during the acute resuscitation phase. Seventh, because the transfusion ratios were categorized into fixed ranges, differences within each category may have influenced the results. For example, patients with a plasma-to-pRBC ratio of 0.51 were included in the reference group (0.5–1), while those with a ratio of 1.5 were included in the statistically significant group (1.5–2).

## Conclusions

This nationwide retrospective cohort study suggested that FFP-to-pRBC and PC-to-pRBC ratios > 1 improved survival in patients with severe blunt trauma, with optimal ranges of 1–1.5 for FFP-to-pRBC and 1.5–2 for PC-to-pRBC. High FFP-to-pRBC ratios were particularly beneficial for phenotype 1 patients with truncal trauma with shock. Nevertheless, further prospective studies are necessary to validate these findings and refine the optimal ratio thresholds for different trauma phenotypes.

## Electronic supplementary material

Below is the link to the electronic supplementary material.

Supplementary Material 1

Supplementary Material 2

Supplementary Material 3

## Data Availability

A summary of the JTDB is available at http://www.jtcr-jatec.org/traumabank/index.htm. The specific data within the JTDB, which support the findings of this study, are accessible through Japan Trauma Care and Research. However, these data are subject to access restrictions, as they were utilised under licence for this study and are, therefore, not available to the public. Please refer to Toru Takiguchi (toru-takiguchi@nms.ac.jp) for data access enquiries.

## Abbreviations

FFP: high fresh frozen plasma
pRBC: packed red blood cells
PC: platelet concentrate
JTDB: Japan Trauma Data Bank
AIS: Abbreviated Injury Scale
ISS: injury severity score
RTS: revised trauma score
TRISS-PS: trauma and injury severity score and probability of survival
CCI: Charlson Comorbidity Index
OR: odds ratio
CI: confidence interval
SD: standard deviation
