# Supplementary material for: Transfusion ratios and survival in severe blunt trauma patients receiving massive transfusion
Source: Sci Rep. 2025 Jul 15;15:25519. doi: 10.1038/s41598-025-11338-7 (PMC12263878; doi:10.1038/s41598-025-11338-7)
Supplement: Supplementary file 1 — Supplementary Material 1 [file 41598_2025_11338_MOESM1_ESM.pptx]

## Slide 1
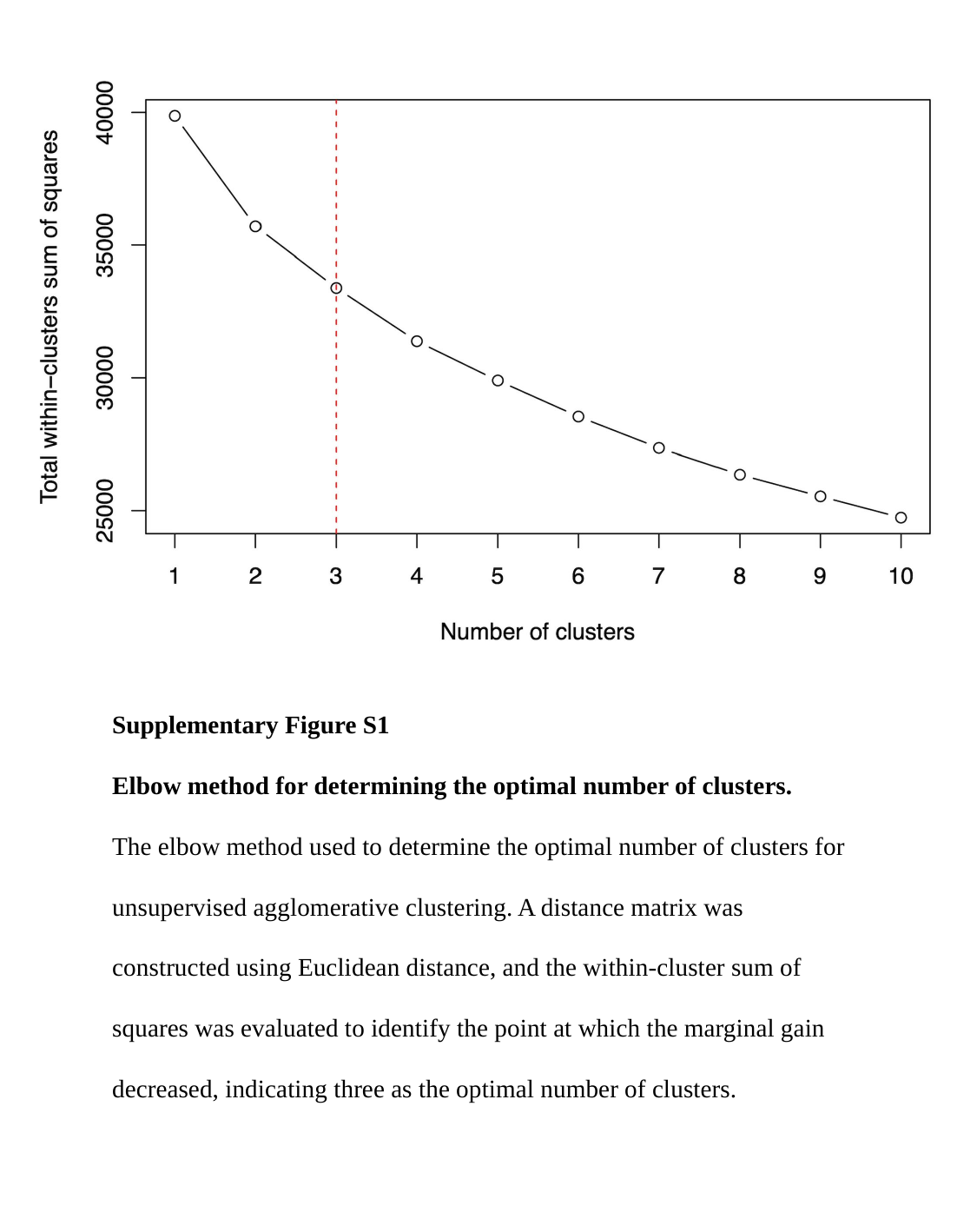

Supplementary Figure S1
Elbow method for determining the optimal number of clusters.
The elbow method used to determine the optimal number of clusters for unsupervised agglomerative clustering. A distance matrix was constructed using Euclidean distance, and the within-cluster sum of squares was evaluated to identify the point at which the marginal gain decreased, indicating three as the optimal number of clusters.
