# Supplementary material for: Transfusion ratios and survival in severe blunt trauma patients receiving massive transfusion
Source: Sci Rep. 2025 Jul 15;15:25519. doi: 10.1038/s41598-025-11338-7 (PMC12263878; doi:10.1038/s41598-025-11338-7)
Supplement: Supplementary file 2 — Supplementary Material 2 [file 41598_2025_11338_MOESM2_ESM.pptx]

## Slide 1
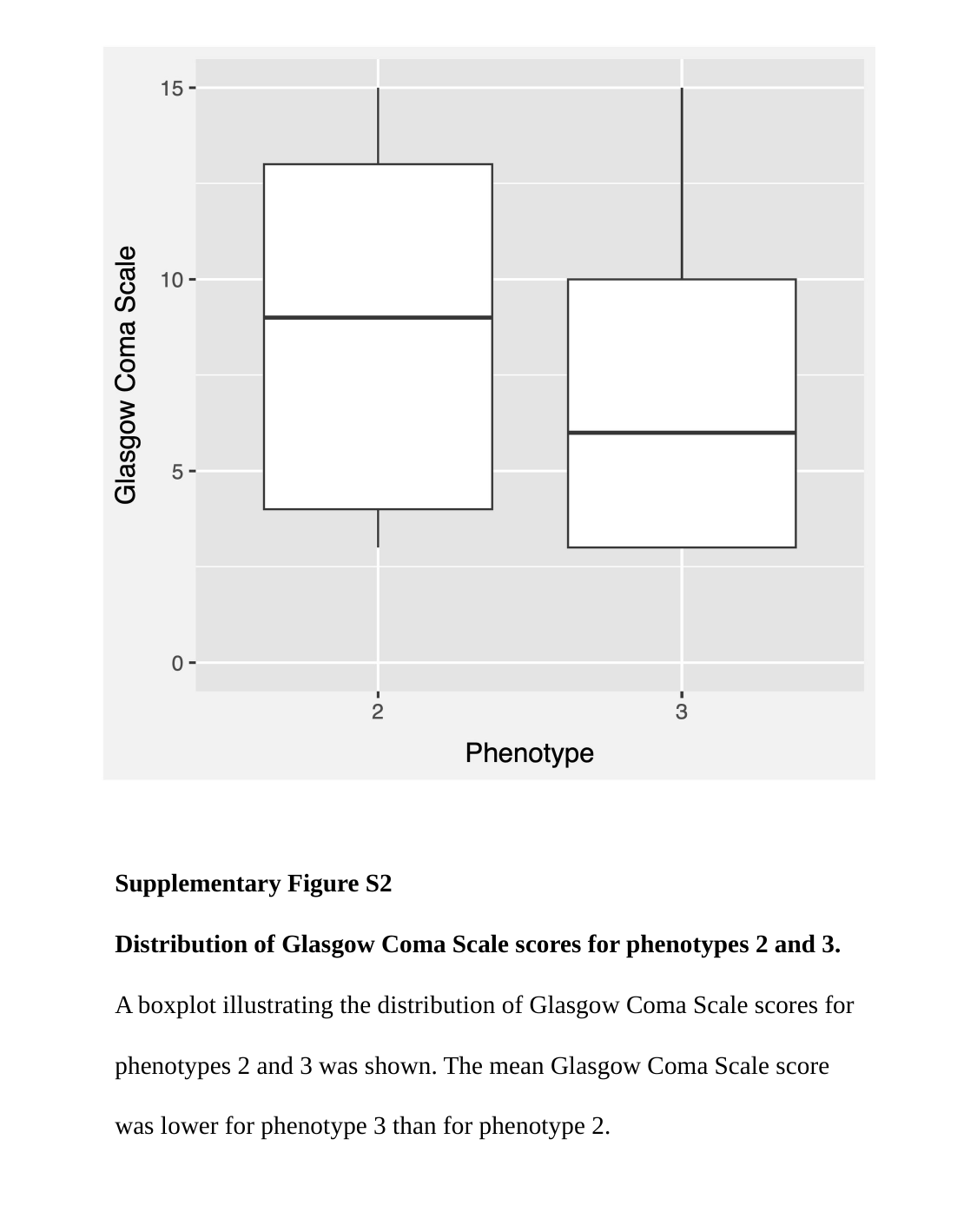

Supplementary Figure S2
Distribution of Glasgow Coma Scale scores for phenotypes 2 and 3.
A boxplot illustrating the distribution of Glasgow Coma Scale scores for phenotypes 2 and 3 was shown. The mean Glasgow Coma Scale score was lower for phenotype 3 than for phenotype 2.
