# Supplementary material for: Transfusion ratios and survival in severe blunt trauma patients receiving massive transfusion
Source: Sci Rep. 2025 Jul 15;15:25519. doi: 10.1038/s41598-025-11338-7 (PMC12263878; doi:10.1038/s41598-025-11338-7)
Supplement: Supplementary file 3 — Supplementary Material 3 [file 41598_2025_11338_MOESM3_ESM.docx]

**Supplementary Table S1.** Total number of FFP and pRBC units for each FFP-to-pRBC ratio category.

| FFP-to-pRBC ratio | Total number of FFP and pRBC units | |
| --- | --- | --- |
| Total |  |  |
| 0 to 0.5 | 24 | (22.9) |
| 0.5 to 1 | 37 | (27.1) |
| 1 to 1.5 | 49 | (31.0) |
| 1.5 to 2 | 48 | (28.5) |
| 2< | 67 | (165.0) |
|  |  |  |
| Phenotype 1 |  |  |
| 0 to 0.5 | 23 | (17.5) |
| 0.5 to 1 | 38 | (28.2) |
| 1 to 1.5 | 49 | (31.8) |
| 1.5 to 2 | 50 | (29.4) |
| 2< | 65 | (156.0) |
|  |  |  |
| Phenotype 2 |  |  |
| 0 to 0.5 | 22 | (12.6) |
| 0.5 to 1 | 39 | (31.7) |
| 1 to 1.5 | 51 | (29.3) |
| 1.5 to 2 | 48 | (23.8) |
| 2< | 50 | (17.3) |
|  |  |  |
| Phenotype 3 |  |  |
| 0 to 0.5 | 30 | (40.9) |
| 0.5 to 1 | 31 | (16.2) |
| 1 to 1.5 | 46 | (28.7) |
| 1.5 to 2 | 44 | (27.7) |
| 2< | 75 | (207.0) |

FFP, fresh frozen plasma; pRBC, packed red blood cells.

Total number of FFP and pRBC units were presented as mean (standard deviation).

FFP-to-pRBC ratio were categorized as follows: 0–0.5 (including 0.5), 0.5–1 (including 1), 1–1.5 (including 1.5), 1.5–2 (including 2), and > 2.

The total number increased with the ratio category in the overall cohort and across all three phenotypes.
